# Usefulness of a psychomotor function test as a cognitive function scale for patients with schizophrenia: A pilot study

**DOI:** 10.1016/j.heliyon.2021.e06719

**Published:** 2021-04-17

**Authors:** Hiroyuki Kamei, Ippei Takeuchi, Yui Yamada, Manako Hanya, Kiyoshi Fujita

**Affiliations:** a Office of Clinical Pharmacy Practice and Health Care Management, Faculty of Pharmacy, Meijo University, Nagoya, Japan; b Department of Psychiatry, Okehazama Hospital Fujita Kokoro Care Center, Toyoake, Aichi, Japan

**Keywords:** Cognitive function, Schizophrenia, Brief assessment of cognition in schizophrenia, Psychomotor function assessment, Compensatory tracking test, Choice reaction time

## Abstract

As cognitive dysfunction due to schizophrenia is strongly associated with patients' social rehabilitation, cognitive functions have been examined as a therapeutic target. Although the Brief Assessment of Cognition in Schizophrenia (BACS) has been used to evaluate cognitive function, it is difficult to administer in routine clinical practice due to its time-consuming nature. Therefore, a novel tool is needed to facilitate the assessment of cognitive function. In the present study, we examined whether cognitive function can be assessed effectively by testing psychomotor function in patients with schizophrenia. Test batteries consisting of choice reaction time (CRT) and compensatory tracking task (CTT) and the BACS were examined in 20 schizophrenic patients to evaluate the correlation between the scales by Pearson correlation coefficient. Of the test batteries, the CRT was significantly correlated with attention functions, a subtest of the BACS (r = -0.506, p = 0.023), and the CTT was strongly correlated with attention functions (r = -0.716, p < 0.001) and working memory (r = -0.633, p = 0.003). A multiple regression analysis was performed to clarify the association between psychomotor function tests and the total BACS score, and peripheral awareness task, a component of CTT, was independently associated with the total BACS score (β = -0.59, p = 0.004) with an R2 of 0.37. Thus, of the psychomotor function tests, the CRT and the CTT are highly useful in assessing cognitive functions in schizophrenic patients. However, no having large sample size in this study is a limitation.

## 1 Introduction

Symptoms of schizophrenia progress slowly and gradually and include the following: 1) positive symptoms, such as hallucinations, delusions, and significant thought disorders, 2) negative symptoms, including a lack of motivation, autistic tendencies, and apathy, and 3) cognitive impairments, such as memory deficits and concentration difficulties (Tandon et al., 2013). Although pharmacological treatment using antipsychotics is the most common therapeutic strategy for schizophrenia, there are currently no effective treatments that specifically target cognitive impairment (Pu et al., 2016). Cognitive function plays an important role in the development of social skills, as well as the ability to develop daily routines (Brüne et al., 2011). Thus, impairments in cognitive function may lead to a wide range of dysfunctions, such as in language, working memory, information processing, attention, and executive function. In addition, previous studies suggested that patients who present with severe cognitive impairment in the early phase of schizophrenia are more likely to develop chronic and severe forms of the disease (Bralet et al., 2008; Hoe et al., 2012). Therefore, the severity of cognitive impairment significantly affects the daily lives of patients and their ability to reintegrate into society.

Patients with schizophrenia have a low level of social and occupational functioning because cognitive impairment significantly affects their working capacity and interpersonal relationships (Bowie et al., 2008). Therefore, the aim of treatment for schizophrenia is the reintegration of patients into society such that they may independently participate in social activities. Improvements in cognitive function have been associated with high-level functional outcomes, including employment (Lexén et al., 2016). Therefore, an accurate assessment of cognitive function is essential for supporting the reintegration of patients with schizophrenia into society. Among several scales of cognitive function, the Brief Assessment of Cognition in Schizophrenia (BACS) is a commonly used scale worldwide (Bralet et al., 2008; Keefe et al., 2004). BACS enables an objective measurement of cognitive impairment, and is used to assess areas of cognitive function, including verbal memory, verbal fluency, working memory, motor speed, attention, and executive function (Keefe et al., 2004). It strongly correlates with other forms of cognitive tests, and is considered to be an excellent tool for evaluating cognitive function in patients with schizophrenia (Keefe et al., 2004). The Japanese version (BACS-J), developed by Kaneda et al. (2007), was also confirmed to be reliable and valid. However, the procedures and instruments used to assess cognitive function in this test are not easily applied in routine clinical practice because they are time-consuming to administer and burdensome for patients. Furthermore, administrators of the tests from each investigative site need to receive special training to become an expert in administration and scoring procedures for test batteries (Keefe et al., 2003). Therefore, a novel tool is needed to assess cognitive function in a simple and objective manner. We previously reported that psychomotor function tests are typically used to evaluate dysfunctions in the central nervous system, such as sedation and hypnosis caused by multiple antihistamines (Kamei et al., 2003, 2012). Test batteries are useful for easily and objectively assessing attention and psychomotor speed using a computer (Hindmarch et al., 2002). Attention and psychomotor speed assessed in our tests are required in cognitive domains because they are strongly associated with vocational outcomes (McGurk et al., 2003).

Therefore, we herein focused on psychomotor function tests (Kamei et al., 2003, 2012), including the choice reaction time (CRT) and compensatory tracking test (CTT), to establish whether they are more applicable than BACS-J to the assessment of cognitive function in patients with schizophrenia.

## 2 Methods

### 2.1 Subjects

The present study included 17 inpatients and 3 outpatients who were diagnosed based on the DSM-Ⅳ criteria for schizophrenia and had been taking antipsychotics for at least 3 months at the Okehazama Hospital Fujita Kokoro Care Center between March 2017 and March 2018. Patients with organic central nervous system disorders, drug or alcohol addiction, severe cognitive deficiency, or mental retardation were excluded. The purpose and significance of the present study were explained to all patients in a written format, and consent was obtained from the patients themselves. The present study was approved by the Institutional Review Board of the Okehazama Hospital Fujita Kokoro Care Center (H29-003).

### 2.2 Study procedures

#### 2.2.1 Baseline and outcome measures

The following baseline measures were collected from each patient: age, sex, years of education, time from diagnosis, and the type and amount of medications currently used. Nine out of 20 patients were on monotherapy with atypical antipsychotics (clozapine: n = 7, olanzapine: n = 2), and 10 were on combination therapy with multiple antipsychotics. The combination of antipsychotics varied among patients. The remaining patient did not take antipsychotics during the study period. The amount of antipsychotics was calculated using the chlorpromazine (CP) equivalent method. Psychiatric symptoms were evaluated using the Positive and Negative Syndrome Scale (PANSS), and severity was assessed by the Clinical Global Impression (CGI) scale. The level of social functioning was evaluated using the Global Assessment of Functioning Scale (GAF), and side effects associated with antipsychotics were assessed by the Drug-Induced Extrapyramidal Symptoms Scale (DIEPSS). Psychomotor function was evaluated using a battery of tests (CRT and CTT) (Kamei et al., 2003, 2012), and cognitive function was assessed by BACS-J (Kaneda et al., 2007).

#### 2.2.2 Test procedures

Psychomotor function tests (CRT and CTT) were performed primarily using computers, and included the assessment of concentration and judgement (Kamei et al., 2003, 2012). The content of each item of the test and how to provide the answers were explained to the patients, and they were given the opportunity to practice the test once prior to the actual test. The practice was performed using training software, which consisted of fewer tasks for CTT than the actual test. Actual measurements were taken after confirming that the patients understood the rules and were fully competent to perform the test. The psychomotor function test and BACS-J were performed on the same day, and lasted for approximately 20 and 50 min, respectively.

### 2.3 Assessment scales

#### 2.3.1 Assessment of psychomotor function

The psychomotor function test is typically used to evaluate dysfunctions in the central nervous system, such as sedation and hypnosis caused by multiple antihistamines. Although subjects may recognize sedative effects, including drowsiness, these effects may also reduce concentration, judgement, and working efficiency without their awareness. Therefore, these effects are used to obtain objective measurements of psychomotor function (Hindmarch et al., 2002). The 2 sub-tests (CRT and CTT) that comprise the psychomotor function test are described below.

##### 2.3.1.1 CRT

CRT is used as an indicator of sensorimotor function and assesses the ability to focus on and react to a critical stimulus (Kamei et al., 2003, 2012). The instrument was equipped with a start button in the middle, as well as 6 reaction buttons aligned in a fan-shape at equal intervals. Each reaction button had red and green lights that were also aligned in a fan-shape, and the green light turned on prior to the red light. After placing the index finger onto the start button, patients were asked to use the green light as a warning in order to react quickly when the red light turned on. Patients were asked to press the reaction button next to the red light as soon as it lit up in order to turn it off. The number of warning green lights gradually increased (1, 3, and 6 light(s) in this order) and decreased (6–3 and then 1 light(s) in this order) during the test. The task was performed 8 times at each level. Therefore, measurements were taken 48 times in a single test. The time between the red light coming on and the finger being released from the starting button was taken as CRT. Low scores reflected a rapid reaction to the task, indicating a high level of attention.

##### 2.3.1.2 CTT

CTT is composed of the following two tasks:

• **Tracking task (TT)**

TT evaluates the ability to react to and track an object (Kamei et al., 2003, 2012). In TT, subjects were asked to track a round object that appeared on the computer screen. The object moved along a straight line in a random manner, and subjects were asked to place the cursor in the middle of the object using a computer mouse and track its movement for 9 min. The distance between the center of the object and the cursor was measured in pixels, and performance was evaluated as the mean distance between the two points for samples randomly selected during the 9-minute test. Low scores indicated the ability to accurately track the object.

• **Peripheral awareness task (PAT)**

PAT was performed at the same time as TT, ensuring that it did not interfere with TT. PAT evaluates peripheral awareness (Kamei et al., 2003, 2012) and lasts for 9 min, similar to TT. In PAT, 100 stimuli (yellow objects) randomly appeared at the 4 corners of the computer screen, and subjects were asked to click on the computer mouse as soon as they saw the yellow object while performing TT. The objects appeared for 3 s at an interval of 3–6 s. The average time interval between the appearance of the object and the click of the mouse was calculated in msec. Low scores indicated high peripheral awareness, reflecting a high level of attention.

The scores of each test were expressed as Z scores relative to healthy volunteers; the average score for healthy volunteers in each age group was considered to be 0, and the scores for study subjects were calculated using the standard deviation (SD). The mean and standard deviation of CRT and CTT scores in normal Japanese individuals were derived from published data (Kamei et al., 2012).

#### 2.3.2 Assessment of cognitive function

BACS-J was performed in the present study. BACS was developed by Keefe et al. (2004), and the Japanese version by Kaneda et al. (2007). BACS-J is used to evaluate cognitive function in schizophrenia patients and comprises 6 sub-tests that assess verbal memory, verbal fluency, working memory, motor speed, attention, and executive function. The BACS sub-tests included in the assessment and their respective tasks are summarized in Table 1. Scores were expressed as Z scores relative to healthy volunteers; the average score for healthy volunteers in each age group was considered to be 0, and the scores for study subjects were calculated using the standard deviation (SD). The mean and standard deviation of BACS scores in normal Japanese individuals were derived from published data (Kaneda et al., 2013). Negative scores in schizophrenia patients indicated lower cognitive function than in healthy volunteers in the same age group. The total score of all 6 sub-tests represented the average Z score for each BACS task.

**Table 1:** Sub-tests for brief assessment of cognition in Schizophrenia Japanese version (BACS-J).

Sub-tests	Tasks
Verbal memory and learning	List learning
Working memory	Digit sequencing task
Motor speed	Token motor task
Verbal fluency	Category instances, Controlled and word association test
Attention and information processing speed	Symbol coding
Executive function	Tower of London

#### 2.3.3 Assessments of psychiatric and other states

The Positive and Negative Syndrome Scale (PANSS) is composed of 30 items: 7 items for positive symptoms, 7 for negative symptoms, and 16 for general psychopathological symptoms. Subjects were evaluated in an interview, and a score between 1 (no symptom) and 7 (most severe) was given to each item. High scores indicate severe symptoms (Krekels et al., 2017). CGI assesses the overall severity of the disease, and is based on physicians’ assessments of a patient. Scores are given between 1 (normal) and 7 (very severe). A high score indicates very severe symptoms (Haro et al., 2003). GAF assesses the severity of symptoms as well as the psychological, social, and occupational functioning of subjects. Scores between 1 and 100 were given in 10-point intervals (Yamauchi et al., 2001). Low scores indicated reduced function in each area.

### 2.4 Statistical analysis

In order to evaluate the applicability of the psychomotor function tests (CRT and CTT) to the assessment of patients with schizophrenia, the outcomes of these tests were compared with the sub-score and total score of BACS, and the outcomes of psychiatric assessments using Pearson's correlation coefficient. A multiple regression analysis was performed to clarify the relationship between psychomotor function tests and the total BACS score, which correlated with most of the components of the psychomotor function tests.

All statistical analyses were performed using SPSS (version 24; IBM Co., Armonk, NY, USA), and p < 0.05 was considered to be significant.

## 3 Results

### 3.1 Patient characteristics

The baseline characteristics and psychiatric symptoms of the 20 patients enrolled in the present study are summarized in Table 2. There were 10 male (50%) and 10 female (50%) patients, with 17 (85.0%) being inpatients and the remaining 3 (15.0%) being outpatients. The age of patients was 45.4 ± 11.7 (mean ± SD) years. On average, patients received 12.2 ± 1.5 years of education, had been diagnosed for 18.5 ± 7.5 years, and were taking 964.3 ± 453.9 mg of CP daily.

**Table 2:** Background and psychiatric symptoms of patients.

Background	Gender	Male	10 (50.0%)
		Female	10 (50.0%)
	Inpatient		17 (85.0%)
	Outpatient		3 (15.0%)
	Age (years)		45.4 ± 11.7
	Years of education (years)		12.2 ± 1.5
	Time from diagnosis (years)		18.5 ± 7.5
	Total dose of antipsychotics CP equivalent (mg/day)		964.3 ± 453.9
Psychiatric symptoms	PANSS (total)		91.9 ± 10.1
	Positive symptoms		22.4 ± 3.0
	Negative symptoms		21.8 ± 2.8
	General psychopathological symptoms		47.3 ± 5.8
	CGI		5.0 ± 1.1
	GAF		38.3 ± 17.6

Values are expressed as the mean ± SD.

CP: Chlorpromazine, PANSS: Positive and Negative Syndrome Scale, CGI: Clinical Global Impression, GAF: Global Assessment of Functioning Scale.

The mean scores for positive, negative, and general psychopathological symptoms measured by PANSS were 22.4 ± 3.0, 21.8 ± 2.8, and 47.3 ± 5.8, respectively, with a total PANSS score of 91.9 ± 10.1. The mean CGI and GAF scores for social functioning were 5.0 ± 1.1 and 38.3 ± 17.6, respectively.

### 3.2 Outcomes of psychomotor function and cognitive function tests

The results from the psychomotor function test battery and BACS in schizophrenia patients are shown in Table 3. The scores for psychomotor functions assessed in the present study were as follows: 8.00 × 10^−5^ ± 1.03 for CRT, -1.00 × 10^−2^ ± 1.03 for CTT-TT (a component of CTT), and 4.00 × 10^−5^ ± 1.03 for CTT-PAT (a component of CTT). In BACS, scores between -0.5 to -1.0 indicate mild cognitive impairment, -1.0 to -1.5 moderate cognitive impairment, and below -1.5 severe cognitive impairment. The mean BACS scores for cognitive function in 20 schizophrenia patients were -2.8 ± 1.4 for verbal memory, -1.2 ± 1.0 for verbal fluency, -2.0 ± 1.4 for working memory, -2.5 ± 1.2 for motor function, -2.3 ± 1.3 for attention, and -2.5 ± 2.4 for executive function, with a total mean score of -2.2 ± 1.0.

**Table 3:** Outcomes of psychomotor function and cognitive function tests.

	Test item	Schizophrenia patients
Psychomotor function tests	CRT	8.00 × 10^−5^ ± 1.03
	CTT-TT	-1.00 × 10^−2^ ± 1.03
	CTT-PAT	4.00 × 10^−5^ ± 1.03
Cognitive function tests	BACS total score	-2.24 ± 1.00
	Verbal memory	-2.78 ± 1.42
	Verbal fluency	-1.22 ± 1.02
	Working memory	-2.04 ± 1.44
	Motor speed	-2.53 ± 1.20
	Attention	-2.34 ± 1.35
	Executive function	-2.53 ± 2.50

Values are expressed as the mean ± SD.

CRT: choice reaction time, CTT: compensatory tracking test, TT: tracking task, PAT: peripheral awareness task, BACS: Brief Assessment of Cognition in Schizophrenia (Japanese version).

### 3.3 Correlation between psychomotor function tests (CRT and CTT) and BACS

The relationships between the results from psychomotor function tests (CRT and CTT) and cognitive function assessed by BACS are summarized in Table 4. The total BACS score for cognitive function negatively correlated with CRT (r = -0.506, p = 0.023), CTT-TT (r = -0.463, p = 0.040), and CTT-PAT (r = -0.609, p = 0.004). The correlation was negative because high CRT, CTT-TT, and CTT-PAT and low BACS scores indicate reduced cognitive function. A correlation was observed between attention in BACS and CRT (r = -0.521, p = 0.019). A correlation was also noted between working memory in BACS and CTT-TT (r = -0.484, p = 0.031). In addition, CTT-PAT was associated with overall BACS scores, and correlated with verbal fluency (r = -0.499, p = 0.025), working memory (r = -0.633, p = 0.003), motor speed (r = -0.486, p = 0.03), and attention (r = -0.716, p < 0.001) in BACS.

**Table 4:** Correlation between outcomes of psychomotor function tests and BACS in all patients.

Psychomotor function tests	BACS						
	Verbal memory	Verbal fluency	Working memory	Motor speed	Attention	Executive function	Total score
CRT	-0.176^a^ 0.459^b^	-0.391^a^ 0.089^b^	-0.422^a^ 0.064^b^	-0.272^a^ 0.245^b^	-0.521^a^ 0.019^b^	-0.286^a^ 0.221^b^	-0.506^a^ 0.023^b^
CTT-TT	-0.101^a^ 0.672^b^	-0.173^a^ 0.465^b^	-0.484^a^ 0.031^b^	-0.257^a^ 0.274^b^	-0.344^a^ 0.138^b^	-0.394^a^ 0.086^b^	-0.463^a^ 0.040^b^
CTT-PAT	-0.409^a^ 0.074^b^	-0.499^a^ 0.025^b^	-0.633^a^ 0.003^b^	-0.486^a^ 0.030^b^	-0.716^a^ <0.001^b^	-0.048^a^ 0.839^b^	-0.609^a^ 0.004^b^

BACS: Brief Assessment of Cognition in Schizophrenia (Japanese version), CRT: choice reaction time, CTT: compensatory tracking test, TT: tracking task, PAT: peripheral awareness task. ^a^ Pearson correlation coefficient, ^b^ p-value.

## 4 Multiple regression analysis with BACS scores as outcome variables

A multiple regression analysis was performed to clarify the relationship between psychomotor function tests and the total BACS score because it correlated with the majority of components in the psychomotor function tests. The total BACS score was defined as the outcome variable, and CRT, CTT-TT, and CTT-PAT scores as explanatory variables (Table 5). CTT-PAT was independently associated with the total BACS score (β = -0.609, p = 0.004) with an R^2^ of 0.37 (Table 5).

**Table 5:** Multiple regression analysis with the total BACS score as the outcome variable.

	Unstandardized coefficient	Standardized coefficient	t-value	p-value
Constant	-2.235		-12.335	<0.001
CTT-PAT	-0.590	-0.609	-3.258	0.004

R^2^ = 0.37.

BACS: Brief Assessment of Cognition in Schizophrenia (Japanese version), CTT: compensatory tracking test, PAT: peripheral awareness task.

## 5 Discussion

Cognitive function assessments play an important role in the accurate and objective evaluation of functional impairment in schizophrenia patients aiming for social reintegration. Due to the relationship between cognitive function tests and psychomotor function tests, we attempted to clarify the applicability of psychomotor function tests for the assessment of cognitive function.

Patients with schizophrenia often have impaired cognitive function, including verbal memory, working memory, attention and alertness, reasoning and problem solving, and information processing, as well as impairments in the functions required for social interactions, such as the ability to recognize facial expressions (Tandon et al., 2013). Cognitive impairments are common and associated with a lack of motivation and the worsening of patient-physician relationships and long-term treatment outcomes. Cognitive function assessed by BACS (Keefe, 2008) was 1.5-2-fold lower in patients with schizophrenia than in healthy volunteers. Patients in the present study had moderately impaired verbal fluency with a Z score of -1.2 as well as severely impaired verbal learning, working memory, motor function, attention, and executive function with Z scores below -2.0. The overall score was -2.2, suggesting that the patient population had moderate to severe cognitive impairment.

We examined the relationship between psychomotor function tests (CRT and CTT) and BACS, and revealed a negative correlation between CRT and the BACS sub-score for attention. This result suggested that positive outcomes in CRT indicate a high level of attention. The correlation between CRT and BACS may be explained by CRT reflecting cognitive function for attention. PAT is a component of CTT and was associated with many of the sub-scores of BACS. PAT negatively correlated with attention and working memory. These results suggest that positive outcomes in PAT indicate high levels of attention and working memory in BACS. CTT consists of 2 tasks performed simultaneously, and PAT reflects the level of attention, as does CRT. Attention is the ability to focus on a target stimulus and maintain that level of attention while the stimulus is being processed. In addition to attention, subjects undergoing PAT will likely require working memory, which is needed to memorize actual tasks, which may explain the relationship between PAT and working memory in addition to attention. We also performed a multiple regression analysis with attention in BACS as the outcome and demonstrated that PAT was independently associated with the BACS score, indicating that PAT and CRT are effective measures of cognitive function. Cognitive function, such as attention and working memory, has a stronger impact on employment than psychiatric symptoms and the time from diagnosis (Kaneda et al., 2009). The magnitude of cognitive impairment has been shown to predict daily living abilities and real-world functioning to a greater extent than psychiatric symptoms (Green et al., 2000). A previous study that examined the relationship between cognitive function and employment in patients with schizophrenia revealed that working memory was a strong factor influencing the employment status and that improvements in working memory resulted in greater social functioning (Green et al., 2000). Therefore, the assessment of cognitive abilities related to daily-living skills or functional capacity is important to facilitate the development of novel therapies and improve daily-living functioning. The extent of on-job support and contact with employment specialists were predicted by the cognitive domains of attention and psychomotor speed (working memory) as well as by the severity of psychotic symptoms (McGurk et al., 2003). Collectively, these findings and the present results demonstrate that attention and working memory are both important functions for patients to reintegrate into society, and their assessment using psychomotor function tests including CRT and CTT may reflect the ability to achieve this goal.

In contrast to interviews in other cognitive tests, psychomotor function tests are simple to conduct because administrators are not required to ask questions to subjects and the tests are performed within a set amount of time. They also do not require specialized skills by administrators and are associated with a low risk of measurement errors. Therefore, they overcome the issues associated with BACS, specifically the need for administrators to obtain BACS certification. In addition, CRT and CTT may be performed within a shorter period of time than BACS. Cognitive function tests that are not time-consuming are preferred because they reduce stress in study subjects and are more efficient.

The present study has several limitations. The results obtained may not be generalizable because they were based on data collected from 20 patients with schizophrenia. Future studies are needed with a larger sample size to clarify the reliability of CRT and CTT measurements. Moreover, although antipsychotics are categorized into first and second generations with no significant differences in terms of their impact on cognitive function (Ayesa-Arriola et al., 2013), further studies are required to assess whether the dose and type of antipsychotics or any other factors influence the outcomes of psychomotor function tests.

Furthermore, we did not compare our psychomotor function tests with other cognitive function evaluations. The Clinical Antipsychotic Trials of Intervention Effectiveness (CATIE) trial showed that 93% of the variance observed in the cognitive composite score was accounted for by 6 tests that required only 35 min to complete (Keefe et al., 2006). Furthermore, in the CATIE trial, the Digit Symbol coding task, which takes less than 5 min to complete, accounted for more than 50% of the variance observed in the cognitive composite score (Gonza'lez-Blanch et al., 2011). Our psychomotor function tests (CRT and CTT) required 20 min to complete. In measurements of cognitive function, it is important to consider not only the balance between brevity and comprehensiveness, but also the characteristics of cognitive function. Our tests are characterized by high sensitivity to attention and psychomotor speed (working memory), which are required to perform the relevant tasks (McGurk et al., 2003). In addition, since our tests use a computer and are easy to operate, they do not need to be performed by a specially trained clinician and the patient also does not require any special training. Additionally, in comparisons with the Screen for Cognitive Impairment in Psychiatry (SCIP), which takes an average of approximately 15 min to complete (Gómez-Benito et al., 2018), our test appeared to be superior for assessing attention and psychomotor speed (working memory) over SCIP, which predicts global cognitive impairment. Therefore, although our tests may be useful, they need to be directly compared with other test methods.

In conclusion, CRT and CTT constitute a battery of tests that objectively and easily assess psychomotor function. The present study demonstrated that CRT and CTT were effective for evaluating higher order cognitive function, such as attention, as well as working memory, which influence social functioning. Therefore, among psychomotor function tests, CRT and the CTT are applicable to the assessment of cognitive function in patients with schizophrenia in real-world clinical settings.

## Declarations

### Author contribution statement

Hiroyuki Kamei: Conceived and designed the experiments; Performed the experiments; Contributed reagents, materials, analysis tools or data; Wrote the paper.

Ippei Takeuchi: Performed the experiments; Contributed reagents, materials, analysis tools or data.

Yui Yamada: Performed the experiments; Analyzed and interpreted the data; Contributed reagents, materials, analysis tools or data.

Manako Hanya: Conceived and designed the experiments; Analyzed and interpreted the data.

Kiyoshi Fujita: Conceived and designed the experiments.

### Funding statement

This work was supported by the Meijo University Budget.

### Data availability statement

Data included in article/supplementary material/referenced in article.

### Declaration of interests statement

The authors declare no conflict of interest.

### Additional information

No additional information is available for this paper.
